# New markers of insulin resistance in polycystic ovary syndrome

**DOI:** 10.1007/s40618-016-0523-8

**Published:** 2016-07-29

**Authors:** K. Polak, A. Czyzyk, T. Simoncini, B. Meczekalski

**Affiliations:** 1 Department of Clinical and Experimental Medicine, Division of Obstetrics and Gynecology, University of Pisa, Pisa, Italy; 2 Department of Gynecological Endocrinology, Poznan University of Medical Sciences, ul. Polna 33, Poznan, Poland

**Keywords:** Polycystic ovary syndrome, Insulin resistance, Markers, Adipocytokines

## Abstract

Polycystic ovary syndrome (PCOS) is the most common endocrine-metabolic disorder in women of reproductive age. The diagnostic criteria include two out of three features: hyperandrogenism, polycystic ovaries on ultrasound and menstrual irregularities (Rotterdam Criteria 2003). PCOS patients are more vulnerable to develop diabetes, cardiovascular diseases and metabolic syndrome. Insulin resistance (IR) is prevalent in women with PCOS independently of obesity and is critically involved in reproductive and metabolic complications of the syndrome. Several tests have been developed to measure IR, some very reliable but complex like the hyperinsulinemic euglycemic glucose clamp and others less precise but easier and less invasive like HOMA-IR. New markers are needed to reach a more reliable assessment of insulin resistance. To date, several surrogate markers have been proposed in the literature to facilitate and improve the determination of IR. Many new proteins are strongly involved with PCOS physiopathology and IR, such as some adipocytokines (adiponectin, visfatin, vaspin and apelin), copeptin, irisin, PAI-1 and zonulin. Many other proteins have been proposed as potential new markers of IR in PCOS, such as resistin, leptin, RBP4, kisspetin and ghrelin, but their role is still controversial. In this review, we provide a short characterization of these new markers, recently studied as indicators of metabolic state.

## Introduction

Polycystic ovary syndrome (PCOS) is a common endocrine disease linked with metabolic complications, and insulin resistance (IR) plays an important role in the development and persistence of this disorder [1]. The gold standard for directly measuring insulin sensitivity in humans is the hyperinsulinemic euglycemic glucose clamp, but it is time consuming, labor intensive, expensive and technically demanding [2]. The homeostatic model assessment of IR (HOMA-IR) is the main method for IR measurement used in scientific studies because it is a simple and noninvasive [3]. Due to the relevance of IR in the setting of PCOS, it would be important for both research and clinical application to identify additional biochemical parameters that may help in identifying IR.

## PCOS and insulin resistance

Polycystic ovary syndrome (PCOS) is the most common endocrine disorder affecting 10–15 % of women in reproductive age. The etiology of the disease is still not totally understood, and the clinical presentation is extremely variable but generally includes clinical or biochemical hyperandrogenism, menstrual disorders (oligo-amenorrhea) and polycystic ovaries on ultrasound.

In the last years, specific diagnostic criteria were developed to allow the diagnosis of PCOS. In 1990 were established the criteria of the National Institute of Health (NIH), followed by ESHRE/ASRM (European Society of Human Reproduction and Embryology/American Society of Reproduction Medicine) criteria established in Rotterdam in 2003. After exclusion of other diseases, the diagnosis of PCOS requires two out of the following three criteria: oligo-anovulation, clinical or biochemical androgen excess, ultrasound polycystic morphology of at least one ovary (at least 12 follicles measuring 2–9 mm in diameter or ovarian volume greater than 10 cm^3^).

In 2011, the Amsterdam ESHRE/ASMR-sponsored 3rd PCOS Consensus Workshop Group identified different PCOS phenotypes, separating the classic phenotype (hyperandrogenism and chronic anovulation, with or without PCO) from those with ovulation disorders and polycystic morphology [4].

The clinical phenotypes present different metabolic risks, and IR is a hallmark of the classic and the ovulatory phenotypes [5]. IR and hyperinsulinemia determine hyperandrogenemia stimulating ovarian theca cells to secrete androgens and increasing LH effect on ovarian androgen production. Both androgens and insulin inhibit SHBG secretion, increasing free and bioactive androgen levels and making clinical androgen excess worse. Moreover, IR is critically involved in the development of metabolic syndrome and cardiovascular disease in PCOS women, that is why its treatment (lifestyle changes, insulin-sensitizing drugs and bariatric surgery) is thought to important both to decrease IR and to alleviate its consequences [6].

Measuring insulin resistance is neither simple nor necessarily accurate. This is why several tests have been developed to quantify this metabolic phenomenon. Some of these tests are very reliable but complex, while others are less precise but more readily usable in clinical research due to simplicity and reduced invasiveness. Hyperinsulinemic euglycemic glucose clamp is the gold standard for directly determining metabolic insulin sensitivity. It consists in a constant insulin infusion resulting in a new steady-state insulin level above the fasting level that enhances glucose disposal in skeletal muscle and adipose tissue and inhibits hepatic glucose production. 20 % dextrose is given to “clamp” blood glucose levels in the euglycemic range. This technique directly measures all body glucose disposal under steady-state insulin conditions, but it is time consuming, labor intensive, expensive and technically demanding [2].

The homeostasis model assessment (HOMA) determines IR and pancreatic β-cell function from basal glucose and insulin (or C-peptide) levels with a simple mathematically derived nonlinear equation (HOMA-IR: fasting insulin (mU/ml) × fasting glucose (mmol/l)/22.5 (normalizing factor)) [3].

The quantitative insulin sensitivity check index (QUICKI) is a simple variation of the HOMA equation that provides a consistent and accurate insulin sensitivity index with positive predictive power and with a significantly better correlation with glucose clamp determinations of insulin sensitivity than other simple surrogates [7]. It is easy, minimally invasive, inexpensive and commonly used in clinical research studies.

The oral glucose tolerance test (OGTT) is a simple test widely used in medical practice to identify glucose intolerance or type 2 diabetes. After an oral glucose load (75 g), blood glucose and insulin concentrations are determined at 0, 30, 60 and 120 min. OGTT provides important information about glucose tolerance, but not insulin sensitivity/resistance per se [8].

The minimal model analysis of the frequently sampled intravenous glucose tolerance test (FSIVGTT) is a simple indirect measurement of insulin sensitivity/resistance. Blood samples are taken for 3 h after an intravenous bolus of glucose administration followed by an insulin infusion. The collected data are analyzed using a computer program MINMOD to provide an insulin sensitivity index [9].

Matsuda index is a simple assessment of insulin sensitivity from the OGTT that provides an excellent approximation of whole-body insulin sensitivity (WBISI = 10,000/√ (fasting glucose × fasting insulin) (mean glucose × mean insulin) [10].

## New markers: structure, action and role in insulin resistance

Although several tests exist to assess insulin resistance, the availability of new markers is highly needed, in the aim to achieve a more reliable assessment of insulin metabolism. To date, a number of new proteins have been proposed as surrogate markers for the assessment of insulin resistance. Below, we provide a short characterization of markers, which have recently been studied as serum/plasma indicators of metabolic state.

### Adipocytokines

Adipose tissue is implicated in the secretion of several hormones such as adiponectin, resistin, leptin, visfatin, apelin, retinol-binding protein 4 (RBP 4), called adipocytokines, that are involved in energy homeostasis and metabolism [11].

Adiponectin is secreted exclusively by mature adipocytes. Decreased adiponectin plasma levels are associated with insulin resistance [12], obesity, type 2 diabetes mellitus and cardiovascular diseases [13]. There are many scientific studies on adiponectin in PCOS patients. Sepilian and Nagamani [14] reported a significant negative correlation between serum adiponectin levels and insulin resistance, with no correlation between BMI and adiponectin in PCOS patients, suggesting that insulin sensitivity seems to be the major determinant of adiponectin levels rather than adiposity.

Panidis et al. [15] observed lower serum adiponectin levels in PCOS patients with insulin resistance (measured by HOMA-IR) than in normal population. Similar findings were observed by Yildiz et al. [16] who demonstrated a relationship between increased insulin resistance and decreased adiponectin levels, suggesting that it could be an adequate marker of insulin resistance and its consequences such as diabetes mellitus and metabolic syndrome.

Resistin is an adipose-derived peptide hormone discovered in 2001 that potentially links obesity and diabetes mellitus [17]. The role of resistin in insulin resistance of PCOS is still controversial. Seow et al. [18] found that hyperinsulinemic PCOS patients and controls had similar serum resistin levels, but resistin mRNA levels were twofold higher in adipocytes from PCOS patients, suggesting a paracrine regulation of insulin resistance. Lewandowski et al. [19] reported a strong negative correlation between adiponectin and resistin levels in PCOS women during OGTT but without a direct correlation with insulin resistance, and they speculated that the adiponectin-to-resistin ratio might help in predicting the cardiovascular risk in these patients. Higher resistin levels were found in PCOS patients by Yilmaz et al. [20], but independently from insulin resistance and BMI. Wang et al. [21] observed significantly higher resistin levels in obese and non-obese PCOS groups than in controls and HOMA-IR had a positive correlation with resistin and a negative correlation with adiponectin. Conversely, Pangaribuan et al. [22] found that none of the 24 PCOS patients in their study presented hyperesistinemia, there was no significant difference in serum resistin concentration between PCOS group and controls, and there was no correlation with BMI and HOMA-IR; instead, they showed that lower adiponectin level was associated with insulin resistance and BMI in PCOS women.

Leptin is a peptide hormone secreted from adipose tissue and plays an important role in food intake and energy homeostasis [23]. Insulin stimulates leptin gene expression, enhancing leptin secretion [24]. The implication of leptin in insulin resistance of PCOS is still unclear. Micić et al. [25] found significantly higher leptin levels in obese PCOS patients compared with non-obese PCOS women and controls with a negative correlation between insulin sensitivity and leptin levels in both PCOS groups. Some years later, Erturk et al. [26] did not reveal significant correlation between leptin and HOMA-IR or hyperinsulinemia, suggesting that leptin levels are related only to obesity. Opposite results were obtained by Pehlivanov and Mitkov [27], who showed a positive correlation between serum leptin levels and insulin resistance, independently of obesity. One possible explanation for this difference is the heterogeneity of the patients, who presented the BMI in the upper limit of normal values. Recently, similar to Erturk’s study, Nasrat et al. [28] found that leptin and BMI were significantly correlated without a significant association with insulin resistance, but they evaluated only PCOS patients without a control group and they did not analyze the molecular mechanisms for further validation. Garruti et al. [29] reported low leptin levels in serum and in follicular fluid in non-obese PCOS patients undergoing in vitro fertilization, with a significant correlation with BMI and insulinemia.

In 2005, Fukuhara and his group isolated a new adipocytokine called visfatin, which expression increases in obesity. Visfatin has an insulin-mimetic effect in mice and in vitro reduces plasma glucose levels [30, 31]. Kowalska et al. [32] reported lower insulin sensitivity in both lean and obese PCOS patients, but increased serum visfatin levels only in lean PCOS women with a negative correlation with insulin sensitivity and a positive correlation with markers of hyperandrogenism, suggesting that obesity may deregulate visfatin expression and that other factors may be involved in this process. Similar findings in non-obese PCOS subjects were observed by Gen et al. [33], who reported also a strong association with total cholesterol, high-density lipoprotein and markers of hyperandrogenism. Later, in a study performed on obese PCOS adolescent girls, Cekmez et al. [34] showed a significant enhancement of HOMA-IR and visfatin and apelin levels and significantly lower adiponectin levels, speculating that adipocytokines can be used as specific markers for insulin resistance. Nevertheless, a recent meta-analysis including 1341 patients indicated visfatin levels were significantly elevated in PCOS subjects, suggesting that visfatin could be a potential biomarker for PCOS, but there was no correlation with insulin resistance, BMI and hyperandrogenism [35].

Visceral adipose tissue-derived serine protease inhibitor (vaspin) is a novel adipocytokine identified in obese diabetic rats, where it improves glucose tolerance and insulin sensitivity [36]. In a small case–control study performed by Tan et al. [37], obese PCOS women had enhanced vaspin mRNA and protein in omental adipose tissue and high plasma vaspin levels, which decreased after treatment with metformin. Positive associations with HOMA-IR and glucose were found, suggesting that high vaspin levels may be a consequence of insulin resistance. Similar results were found by Koiou et al. [38]. In their study, serum vaspin levels were higher in both lean and obese PCOS patients than in controls. QUICKI was lower in PCOS subjects, suggesting that insulin resistance and obesity enhance vaspin levels. However, treatment with metformin, diet and weight loss appeared to not affect serum vaspin concentrations.

Apelin is a peptide isolated from bovine stomachs, but it is expressed in several other organs and also in visceral and subcutaneous tissues [39]. Apelin was found to be higher in PCOS patients by Gören et al. [40] but without a significant correlation with HOMA-IR. Olszanecka-Glinianowicz et al. [41] reported an inverse association between apelin and glucose, insulin and HOMA-IR values, supporting the role of apelin in the regulation of insulin sensitivity. Apelin levels were higher in non-obese PCOS patients, suggesting a compensatory mechanism for metabolic consequences of insulin resistance. Different from Cekmez’s study [34], lower serum concentrations of apelin were found in PCOS subjects by Altinkaya et al. [42] with positive correlation with BMI, insulin, HOMA-IR, triglyceride and free testosterone, speculating that apelin can be used as a marker for insulin sensitivity. Conversely, Sun et al. [43] observed significantly enhanced apelin concentration in PCOS patients with positive association with BMI and HOMA-IR; treatment with drospirenone-ethinylestradiol plus metformin improved insulin resistance and apelin levels. Discrepant findings among the published studies may be attributed to the differences in ethnicity, age, study design, sample size, genetic characteristics of populations, and assessment methodology.

The data concerning serum retinol-binding protein 4 (RBP4), an adipocytokine involved in systemic insulin resistance, are inconclusive. Weiping et al. [44] found elevated RBP4 levels in PCOS patients regardless of obesity with negative correlation with insulin sensitivity, suggesting that this protein could be an important link between adipose tissue and insulin resistance in PCOS. In line with this study, Möhlig et al. [45] observed that elevated RBP4 levels were significantly associated with insulin resistance. RBP4 levels were higher in women suffering from metabolic syndrome or impaired glucose metabolism, but ROC curve analysis showed that plasma RBP4 does not appear to be a clinically useful parameter to predict metabolic consequences in PCOS women. On the other hand, Hanh et al. [46] noted that RBP4 were associated with obesity but not with indices of insulin resistance and they found no differences in RBP4 concentrations between PCOS patients and controls, concluding that its levels are not influenced by PCOS per se. No correlation with insulin resistance was found also by Hutchison et al. [47], who noted that RBP levels did not change after treatment with metformin or oral contraceptives, which reduced and enhanced insulin resistance, respectively, concluding that RBP4 cannot be used as a marker of insulin resistance in PCOS. Cohort size, the choice of methodology for the evaluation of insulin resistance, selection bias or methodological differences in RBP4 measurements could contribute to obscure this relatively small impact on IR observed. Recently, contrarily to previous studies [45, 46], Olszanecka-Glinianowicz et al. [48] reported significantly higher RBP4 levels in normal weight PCOS compared to obese PCOS subjects and significantly lower concentrations in normal weight controls compared to obese controls, with a negative correlation between RBP4 and HOMA-IR or insulin concentrations, hypothesizing a compensatory effect in the negative feedback between RBP4 and insulin resistance to prevent deterioration in obese PCOS patients.

### Kisspeptin

In the literature, there are limited data about kisspeptin changes and hormonal and metabolic diseases. Kisspeptin, also called metastin, is a peptide produced by KiSS-1 gene involved in puberty activation and is the key stimulating factor of GnRH pulsatile secretion during the ovulation [49]. Panidis et al. [50] found lower kisspeptin serum levels in PCOS women than in controls, and they reported significantly higher kisspeptin levels in normal weight PCOS subjects than in overweight or obese women with the disease with negative correlation with BMI, androgens, fasting insulin levels and HOMA-IR, suggesting that insulin resistance is linked to decreased kisspeptin levels. In this study, the number of control women was relatively small and no lean healthy volunteers were involved. Therefore, it is too soon to draw definite conclusions. Different findings were reported by Jeon et al. [51] and Yilmaz et al. [52], who observed that kisspeptin levels were significantly increased in normal weight and obese PCOS women compared with controls with positive correlation with androgenic profile but without significant association with insulin resistance, suggesting that kisspeptin can be used as a specific marker for hyperandrogenism in PCOS. Further studies are necessary to elucidate the role of kisspeptin in metabolic consequences in PCOS.

### Copeptin

To date, few studies were performed to assess the connection between plasma copeptin levels and metabolic risk in PCOS. Saleem et al. [53] performed a multicenter, community-based study on 1293 African-Americans and 1197 non-Hispanic whites to investigate the association of copeptin, the C-terminal pro-vasopressin fragment, with insulin resistance and presence of metabolic syndrome. In both groups, copeptin levels were higher in participants with metabolic syndrome with positive correlation with insulin levels and HOMA-IR and independent association with adiposity and dyslipidemia, suggesting that copeptin may be a novel marker of insulin resistance and metabolic syndrome. Karbek et al. [54] found enhanced copeptin levels in PCOS patients positively associated with fasting insulin, HOMA-IR, androgenic profile, triglycerides and carotid intima media thickness, indicating that copeptin may play an important role in cardiometabolic consequences in PCOS. Similar findings were observed by Taskin et al. [55], who reported that enhanced copeptin levels in obese PCOS women were associated with insulin resistance and obesity.

### Irisin

Irisin is a novel myokine recently identified in humans and mice involved in brown-fat-like development of white fat, energy expenditure, weight loss and improved glucose tolerance [56]. The relationship between irisin and insulin resistance is still not totally elucidated, and the regulation of this myokine has yet to be demonstrated. Li et al. [57] demonstrated that irisin levels were significantly higher in PCOS subjects than in controls and in overweight and obese patients than in lean women in both groups. Significant positive correlation between circulating irisin with insulin resistance and dyslipidemia was found. They studied the effect of hyperinsulinemia on irisin levels with the hyperinsulinemic euglycemic glucose clamp: At the beginning of the assay, short-term hyperinsulinemia determined a rapid decrease of irisin concentrations in both groups, and therefore, during the steady state of the test, its levels were constant, suggesting that elevated irisin concentrations in PCOS patients may be a consequence of metabolic stress. After metformin therapy, authors observed significantly decreased irisin levels and improved insulin resistance in PCOS women. Recently, similar results were obtained by Li et al. [58], who noted significantly increased irisin levels in overweight and obese PCOS women with high free androgen index (FAI) with an evident increase in insulin resistance and hyperandrogenemia, suggesting that elevated irisin levels predict insulin resistance, metabolic syndrome and hyperandrogenemia.

### Ghrelin

Ghrelin is a multifunctional peptide hormone secreted principally in the stomach. It stimulates several biological functions including food intake, glucose release, cell proliferation and reproduction [59]. The first study assessing the relationship between ghrelin levels and insulin resistance in PCOS was performed by Schöfl et al. [60], who compared PCOS women with healthy controls and gastrectomized subjects. In this study, ghrelin levels were lower in insulin-resistant patients and were similar to the levels found in gastrectomized women; in insulin-sensitive PCOS patients, ghrelin concentration was comparable to controls. A significant correlation between ghrelin levels and insulin resistance was found in PCOS women; treatment with metformin improved insulin sensitivity and enhanced serum ghrelin levels, suggesting that its reduction in PCOS may be a cause or a consequence of insulin resistance. Lower ghrelin levels in PCOS patients were also found by Mitkov et al. [61], with a strong negative association with clinical and hormonal indices of insulin resistance. These data are in conflict with Houjeghani’s et al. [62] results, who did not observe significant difference in ghrelin concentration between PCOS patients and control group, without correlation with BMI and insulin resistance. Similar results were obtained recently by Cassar et al. [63], who reported that ghrelin and other biomarkers were strongly correlated with obesity rather than PCOS per se and that only PAI-1 may be a predictor of insulin resistance independently of PCOS status. This discrepancy of results may be explained by confounding factors, such as body weight, fat mass, age, hormonal status, and severity of disease. Ghrelin may play an important role in the development of PCOS and metabolic consequences, but to date too much heterogeneity was identified and further studies are necessary to establish the exact relationship between ghrelin levels and PCOS.

### PAI-1

Plasminogen activator inhibitor-1 (PAI-1), a glycoprotein involved in the coagulation system, seems to play an important role in PCOS and in its metabolic consequences. Sampson et al. [64] reported increased PAI-1 serum concentrations in non-obese PCOS patients with a significant strong association with hyperinsulinemia. Similar findings were observed by Orio et al. [65] and Tarkun et al. [66], suggesting that increased PAI-1 levels may be a predictor factor in cardiovascular risk in PCOS women. Recently, as mentioned before, Cassar et al. [67] found that PAI-1 was the only biomarker linked to insulin resistance in PCOS subject, with high accuracy to predict IR (>70 %), while other biomarkers investigated in the study (ghrelin, visfatin, leptin, C-protein) were more strongly correlated with BMI rather than PCOS status.

### Zonulin

Zonulin is a novel eukaryotic protein that reversibly regulates gut permeability opening intestinal tight junctions and seems involved in the pathogenesis of autoimmune diseases [67]. The association between zonulin and metabolic disorders has been recently recognized. Moreno-Navarrete et al. [68] investigated the relationship between this protein and obesity and insulin resistance, and they found that zonulin levels appear to be significantly increased in obese and glucose-intolerant subjects with a positive correlation with insulin resistance and inflammatory markers such as IL-6. To date, there is only one study that investigates serum zonulin levels in PCOS patients. Zhang et al. [69] reported significantly elevated zonulin levels in women with the disease compared to controls and demonstrated a strong correlation with insulin resistance, obesity, dyslipidemia and severity of menstrual disorders, speculating that increased gut permeability may alter the intestinal barrier determining an inflammatory state that leads to insulin resistance. Further studies are necessary to confirm the role of zonulin and alterations of intestinal permeability in the physiopathology of PCOS.

## Conclusions

With this article, we provide a short characterization of a variety of methods used for the determination of insulin resistance/sensitivity in PCOS patients. The gold standard for direct measurement of insulin sensitivity is the hyperinsulinemic euglycemic glucose clamp technique, but HOMA-IR and QUICKI are the most used methods in the scientific literature [3, 7]. Many new proteins are now surfacing as potential new markers of insulin resistance in PCOS. In the scientific literature, we found a strong connection between adipocytokines (in particular adiponectin, visfatin, vaspin and apelin), copeptin, irisin, PAI-1 and zonulin with insulin resistance and PCOS physiopathology, while the role of other proteins (such as resistin, leptin, RBP4, kisspetin and ghrelin) is still controversial. Many scientific articles tried to use surrogate markers to simplify the assessment of insulin resistance, anticipate the diagnosis of metabolic and cardiovascular consequences in PCOS, improve the use of medical resources, and reduce costs and side effect. Further studies are necessary to better clarify the role of these proteins with insulin resistance in this syndrome.
